# Will tourism cultural experience affect tourist citizenship behaviors? A case of the “Village Basketball League” in Taijiang County, Guizhou Province

**DOI:** 10.1371/journal.pone.0326613

**Published:** 2025-07-16

**Authors:** Yuxin Yang, Yuting Lin, Chen Qiu, Youcheng Chen, Shuisheng Fan, Zhidan Chen, Yongqiang Ma

**Affiliations:** 1 College of Digital Economy, Fujian Agriculture and Forestry University, Quanzhou, China; 2 Business School, Quanzhou Normal University, Quanzhou, China; 3 College of Rural Revitalization, Fujian Agriculture and Forestry University, Fuzhou, China; Zhongnan University of Economics and Law, CHINA

## Abstract

In the era of the experience economy, the tourist experience has become a key factor in the development of tourist destinations. Unlike traditional sightseeing experiences, tourism cultural experiences allow for engagement in cultural activities at the destination, thereby fostering a deeper emotional connection with the local community, which subsequently influences tourists’ citizenship behavior. The “Village Basketball League” in Taijiang County, Guizhou Province is characterized as a form of tourism cultural experience with high integration and strong involvement. This study presents the “Village Basketball League” in Taijiang County as a case study. Grounded in SOR (Stimuli-Organism-Response) theory and involvement theory, this study explores the influencing factors of tourist citizenship behaviors from the perspective of the integration of culture and sports and constructs a structural equation model that includes tourism cultural experience, tourists’ involvement, flow experience, and tourist citizenship behaviors. Data analysis was performed using SmartPLS 4.0 on questionnaires completed by 314 tourists. The results indicate that: (1) The tourism cultural experience positively affects tourist feedback behaviors, tourist recommendation behaviors, and flow experience; (2) Flow experience positively influences tourist feedback behaviors and recommendation behaviors; (3) Flow experience serves as a mediator between the tourism cultural experience and both tourist feedback behaviors and tourist recommendation behaviors; (4) Tourists’ involvement negatively moderates the predictive effect of tourism cultural experience on flow experience. The research findings offer valuable insights for decision-making regarding the development of tourism destinations and offer guidance for tourism development and management.

## 1 Introduction

With societal advancement and enhanced living standards, tourists’ expectations of destinations have evolved beyond basic satisfaction with scenic attractions and services to prioritize spiritual and cultural experiences [1]. According to the report of the World Tourism Organization (UNWTO), cultural tourism accounts for about 40% of the global tourism market, which is one of the fastest-growing segments [2]. An increasing number of destinations are strategically leveraging indigenous cultural assets and festivals [3], offering immersive experiential opportunities while establishing new paradigms for destination value creation. The “Village Basketball League,” which gained popularity in recent years, exemplifies the integration of culture, sports, and tourism. Originating as a grassroots basketball tournament in Taipan Village, Taijiang County, Guizhou Province, its name reflects the villagers’ passion for basketball, signifying a “rural version basketball league.” Compared with events such as marathons and World Cup football matches, “Village Basketball League” in Taijiang County has a more significant effect on local culture inheritance, embedding Miao ethnic songs and dances, Lusheng performances and intangible cultural heritage exhibitions into the event process to form an immersive experience. Evolving from a local sporting event to a nationwide phenomenon encompassing over 2,000 counties in 31 provinces, autonomous regions, and municipalities, the “Village Basketball League” has transcended its initial purpose, emerging as a vital platform for showcasing local cultures with high integrality and strong engagement. This has contributed to the advancement of the tourism industry across various regions.

In the context of cultural tourism emerging as a prominent trend, tourists whose expectations regarding cultural experiences are fully met tend to allocate greater expenditures towards additional products and services offered by the destination. Furthermore, tourists’ perceptions of the cultural experience at the destination serve as a critical factor influencing their evaluations and behavioral choices [4,5]. Previous scholarship has systematically examined the antecedents and outcomes of memorable tourism experiences [6,7], establishing distinctive experiences as foundational elements for destination competitiveness and sustainability. However, existing research remains predominantly focused on traditional tourism scenarios including ancient towns and ecotourism [8,9], with limited exploration of event-driven cultural-tourism symbiosis mechanisms. As primary co-creators of tourism value, tourists’ attitudes and behaviors towards destinations significantly influence the long-term development of the local tourism industry. Consequently, deciphering the drivers of tourist citizenship behaviors becomes imperative for destination management organizations. Tourist citizenship behavior is defined as the actions exhibited by tourists based on their tourism experiences that contribute to the development of tourism destinations [10]. Currently, abundant research has been conducted on the factors influencing tourist citizenship behaviors. Existing studies have mainly explored the influence of tourists’ personal factors on tourists’ citizenship behavior, for example, Shafiee et al.‘s study found that factors such as tourists’ engagement and social interactions positively influence tourists’ citizenship behavior through the mediating role of relationship quality [11]. Bi and other scholars, based on the social exchange theory and cognitive appraisal theory, revealed that host-tourist interactions shape citizenship behaviors through experiential value and place attachment [12]. In addition, destination-level determinants have emerged as critical antecedents of tourist citizenship behaviors. Zhang et al. established a dual mediation pathway whereby destination social responsibility enhances tourist citizenship behaviors sequentially through destination reputation and destination identification [13]. Rather et al. found that destination brand reputation has a significant positive impact on tourists’ affective, cognitive, and behavioral participation through the lenses of signal theory and social exchange theory,indirectly driving tourists’ citizenship behavior [14]. A number of scholars have also explored the influence mechanism of tourists’ behavioral intentions from the perspective of tourism experience [15,16]. However, there is still a lack of systematic research on how destination cultural experiences influence tourist citizenship behaviors. Most of the existing studies start from a single tourism perspective and focus on tourism experience, destination attributes, and individual characteristics of tourists, paying insufficient attention to factors specific to the process of cultural and tourism integration. In the process of participating in the “Village Basketball League” activities, tourists can experience the competitive atmosphere and gain a deeper understanding of the local traditional culture. However, what is the influence mechanism of tourism cultural experiences on tourists’ behavior, and does such a mechanism exist? How do tourist destinations influence tourists’ behavior through culture? These questions require further empirical research, in which the potential mediating and moderating mechanisms between cultural experience and behavior are not yet clear.

In recent years, more and more scholars have applied the SOR (Stimulus-0rganism-Response) theory to the field of tourism research to study tourists’ behavior. When tourists experience the local culture in the context of the “Village Basketball League,” they are easily attracted and immersed in cultural elements such as events and festivals. This immersion facilitates a flow experience, leading tourists to exhibit destination-friendly behaviors, including feedback and recommendations, thereby contributing to the formation of the “Stimulus-Organism-Response” pathway. Accordingly, based on the SOR theory and the case of Guizhou “Village Basketball League”, this paper introduces the flow experience as a mediating variable in order to systematically explore the influence of tourism cultural experience on tourist citizenship behaviors. In addition, based on the involvement theory and the characteristics of the “Village Basketball League” tournament, this paper introduces the degree of tourists’ involvement as a moderating variable to further clarify the influence of tourism cultural experience on flow experience. The purpose of this paper is to explore the influence of tourism cultural experiences on tourist citizenship behaviors from the perspective of culture and tourism integration. On one hand, it aims to verify the relationships and mechanisms among these variables; on the other hand, it seeks to fill the research gap concerning tourism cultural experiences and tourist citizenship behaviors. At the theoretical level, this paper for the first time integrates tourists’ involvement, cultural experience, flow experience, and citizenship behaviors into the SOR theoretical framework. It systematically reveals the multi-level driving pathways of tourist behavior within the context of cultural, sports, and tourism integration, thereby expanding the explanatory power of the SOR theory in cultural scenarios. Additionally, unlike previous S-O-R studies that focused on the direct drive of external stimuli on behavior, this research introduces involvement as a moderating variable. This approach reveals the dynamic boundary conditions under which cultural stimuli are transformed into individual psychological states, which helps to enrich the theoretical framework of tourist behavior research. At the practical level, this study advances beyond traditional tourism research focused on villages and tourist attractions. It reveals a novel model for guiding regional tourism development through activities and events from the micro level. This study also provides strategic support for promoting the “Village Basketball League” as an event tourism model to the whole country from a “sports + culture” perspective, which has reference value for marathon tourism and other similar event tourism activities. Furthermore, it provides a theoretical basis for tourism destination managers to develop strategies, aids local rural revitalization, and supports cultural heritage preservation. This is significant for tourism destinations aiming to leverage cultural characteristics and enhance local tourism value.

## 2 Literature review and hypotheses development

### 2.1 The Stimulus–Organism–Response theory

The SOR (Stimulus-Organism-Response) theory originates from behavioral psychology, emphasizing that external environmental stimuli induce behavioral responses by affecting an individual’s internal mental state [17]. In tourism research, the SOR framework is widely used to explain the formation mechanisms of tourist behavior. Among them, stimulus mainly refers to the interaction, social media publicity, destination environment image, and tourism experience related to the tourist destination [18–20]. Organism mainly refers to tourists’ perception, emotional state, flow experience and psychological identity with the tourist destination [21–23], Response mainly refers to tourists’ behavioral reactions to the tourist destination, including tourism intention, revisiting intention, environmental responsibility behavior [21,24,25]. In addition, research conducted by Liu et al. further demonstrates that the SOR model serves as an effective theoretical framework for exploring tourists’ psychological processes in cultural heritage tourism [26]. The above studies show that tourism cultural experience, flow experience, and tourist citizenship behaviors are appropriate research variables within the context of the SOR model; yet, few investigations have employed SOR theory to examine the interconnections among these variables. Based on the SOR model and situated cognition theory, Wen et al. examined a series of performances conducted during festivals at various heritage sites as subjects of their research. They proposed that the co-development of festivalscape attributes could be transformed into stimuli that induce favorable tourism experiences, thereby enhancing affective responses and loyalty to both events and heritage sites [27]. Using the SOR theory, Chen et al. explored the implementation of AR technology in cultural heritage tourism and proposed that AR technology experience, as a form of cultural experience, positively influences tourists’ heritage-responsibility behaviors [28].

Although SOR theory is widely used in cultural tourism studies, in specific cultural contexts, such as the “Village Basketball League” tourism where basketball matches are intertwined with folk culture, tourist behavior may be influenced by factors like tourism involvement, which the traditional SOR model may overlook. Considering that tourist citizenship behaviors and their antecedents within the integration of culture, tourism, and sports remain largely unexplored, this study utilizes the SOR model to analyze how tourism cultural experience, as a representative stimulus, influences an individual’s flow experience state and thereby leads to positive intentional responses. By incorporating moderating variables such as tourists’ involvement and contextualized stimulus design, it demonstrates that the effect of cultural stimuli is moderated by the extent of individual participation and the type of the activity, thereby enhancing the explanatory power of the SOR theory for culture-driven tourist behavior. Specifically, this study believes that the synergistic effect of sports events and cultural activities such as the inheritance of intangible cultural heritage skills, constitutes a tourism cultural experience, promotes the flow experience of tourists, and makes them more immersed in the “Village Basketball League” event, generating a sense of pleasure. Through the intermediary role of “flow experience,” cultural identity is enhanced, altruistic motivation is stimulated through emotional engagement, and citizenship behaviors benefit the tourist destination. Finally, the progressive chain of “stimulus-psychological transformation-behavior” is constructed.

### 2.2 Tourism cultural experience and tourist citizenship behaviors

With the rise of the experience economy, tourism is increasingly being experienced by tourists in ways that extend beyond traditional sightseeing. Based on the SOR model, virtual travel experiences are regarded as external stimuli by some scholars, and their impacts on on-the-spot travel intentions are discussed. Consequently, this study also tries to select the stimulus variables of the travel experience model to conduct research [20]. Numerous scholars have investigated the dimensions of tourism experience. Among these scholars, Stanovčić and colleagues argue that focusing solely on the comprehensive impact of tourism experience on behavior may overlook the significance of specific dimensions. They propose studying the influencing factors of tourist behavior from the specific dimensions of cultural tourism experience [4]. As tourism cultural experience constitutes a significant aspect of the overall tourism experience, and given that cultural tourism and sports tourism are currently prominent topics, considerable attention has been drawn from scholars both domestically and internationally. Therefore, this study focuses on the integration of culture, sports and tourism, examining the dimensions of tourism cultural experience.

In the stimulus-organic-response theory, the term “reaction” typically refers to behavioral responses exhibited by individuals. In light of this, tourist citizenship behavior is introduced in this paper as the outcome variable of the study. Currently, abundant research findings exist regarding the dimensions of tourist citizenship behavior. Among these scholars, some draw from Groth’s classification of customer citizenship behavior, asserting that tourist citizenship behavior comprises three dimensions: help, feedback, and recommendation [29]. Additionally, Liu’s research indicates that tourist citizenship behavior can be categorized into two types: tourist-oriented behavior, which primarily assists other tourists in enhancing their experiences at the tourist destination; and destination-oriented behavior, which primarily encompasses actions that benefit the tourist destination, such as providing suggestions or feedback and recommending the destination to others [30,31]. Given that the primary objective of this study is to provide suggestions for tourist destinations, only feedback behavior and recommendation behavior are selected to represent tourist citizenship behavior.

Kim’s research indicates that experience influences individual behavioral intentions [32]; specifically, cultural experiences during tourism affect tourists’ feedback about the destination and their willingness to recommend it. Lee et al. found that there is a positive correlation exists between the quality of tourist experiences and the behavioral intentions of tourists [33]. Therefore, with the improvement of the quality of tourism experience in the destination and the enhancement of the perceived cultural atmosphere, more and more tourists are willing to provide feedback and recommend the destination. According to the research conducted by Cheng and Chen, tourists’ emotional experiences positively influence their behavioral intentions; thus, emotions arising from cultural experiences have a decisive impact on their tourism behaviors [34]. In discussions regarding the relationship between unforgettable travel experiences and tourists’ behavioral intentions, some scholars have found that tourists’ willingness to revisit and recommend destinations through word of mouth positively correlates with their sense of experience. The more unforgettable the experience, the stronger the behavioral intentions of tourists become [35]. Additionally, Xu and other scholars have noted that positive experiences during travel prompt tourists to engage in environmentally responsible behaviors [36]. Based on these findings, the following hypotheses are proposed in this study:

*H*1a: Tourism cultural experience has a significant positive impact on tourist feedback behaviors.

*H*1b: Tourism cultural experience has a significant positive impact on tourist recommendation behaviors.

### 2.3 The mediating role of flow experience

In existing studies, flow experience has been examined by many scholars as an emotional factor of the organism (O) within the SOR theoretical model [37,38]. This indicates that flow experience represents a significant research perspective; therefore, this variable is included in the present study. Flow experience was first introduced by the scholar Csikszentmihalyi, primarily indicating that an individual experiences happiness while engaged in an activity or environment, becoming immersed and disregarding the surrounding context. The value of tourism experiences influences the attainment of tourists’ happiness [39], subsequently affecting their flow experiences. Recent studies have indicated that, in comparison to food and common goods, novel experiences and valuable products offered by tourist destinations attract tourists’ attention more effectively, thereby enhancing their overall happiness [40]. Pleasure and concentration are prominent characteristics of the flow experience. Therefore, the culture of the tourist destination, as a distinctive experience, can enhance tourists’ flow experiences. Wu et al. investigated indigenous tourism experiences and found that tourists tend to feel happiness and fulfillment when participating in indigenous cultural activities in these areas, indicating that cultural experiences in tourism may influence flow experiences [41]. Additionally, studies have indicated that tourists’ perceptions of soundscapes at tourist destinations can positively influence flow experiences, suggesting that tourists are more likely to experience flow when engaging with cultural scenes, such as distinctive handmade crafts and festival activities [8]. As a subjective individual experience, flow experience is easily influenced by the cultural atmosphere perceived by tourists during their travels. Therefore, the following hypothesis is proposed:

*H*2: Tourism cultural experience has a significant positive impact on flow experience.

Many studies have demonstrated that flow experiences induce positive behaviors. Akçakese indicated that the positive emotions generated by flow experiences influence individuals’ environmental behaviors; specifically, the stronger the sense of flow experience, the more likely individuals are to engage in tourist citizenship behaviors that protect the environment and respect nature [42]. Some scholars have proved in their study that the improvement of flow experiences can foster tourists’ loyalty to the destination [43], thus promoting tourists’ feedback behaviors and recommendation behaviors. At the same time, research by Back et al. demonstrates that tourists’ flow experiences at tourist attractions significantly impact their willingness to revisit and recommend [44]. Generally, individuals in a flow state exhibit a greater sense of participation than ordinary tourists and are more inclined to deeply understand the destination and provide feedback. Accordingly, the following hypothesis is presented:

*H*3a: Flow experience has a significant positive impact on tourist feedback behaviors.

*H*3b: Flow experience has a significant positive impact on tourist recommendation behaviors.

In tourism research, flow experience is frequently employed as a mediating variable. For example, Li et al. examined the impact of restorative environmental perceptions mediated by flow experience on tourists’ revisit and recommendation behaviors [45]. Research by Karasakal et al. demonstrates that flow experience plays an intermediary role between destination attribute perception and tourist satisfaction [46]. The triggering of flow experiences often depends on the uniqueness of the situation and the emotional involvement of the participants. The “Village Basketball League,” an event integrating rural culture and sports competition, serves as a potent cultural stimulus (S) through its authentic cultural context, encompassing spontaneously organized competitions by villagers, traditional song and dance performances, and local food culture, encouraging tourists to enter a flow state (O) characterized by high concentration and altered time perception, and thus willing to make suggestions on the defects of the tourist destination. Furthermore, they are likely to recommend the destination to others (R). Based on this analysis and the aforementioned assumptions, it is posited that flow experience serves as a bridge between tourism cultural experiences and tourists’ feedback and recommendation behaviors. Accordingly, the following hypothesis is presented:

*H*4a: Flow experience plays an intermediary role in tourism cultural experience and tourist feedback behaviors.

*H*4b: Flow experience plays an intermediary role in tourism cultural experience and tourist recommendation behaviors.

### 2.4 The moderating effect of tourists’ involvement

In recent years, the theory of involvement has been widely applied by scholars in the field of tourism, with the degree of involvement being a critical component of the theory. The tourist involvement refers to the extent of tourists’ investment in tourism, specifically the psychological state encompassing their interest, motivation, and emphasis on tourism products or activities [47]. It has been suggested by some scholars that tourism involvement is a significant factor influencing the sense of experience. For most tourists, a high degree of involvement results in increased enjoyment derived from tourism activities, facilitating immersion and stimulating the generation of flow experiences [48]. In addition, it has been noted by some scholars in their research that tourism involvement serves a regulatory role between self-harmony and destination attitude [49]. Tourists exhibiting high levels of tourism involvement are likely to have enhanced travel experiences and are more inclined to appreciate and identify with local culture. Following active participation in cultural experiences, tourists are likely to experience greater satisfaction with the destination, which can enhance their sense of flow experience. A high degree of tourist involvement indicates that tourists are both interested in and enthusiastic about the activities offered at the tourist destination, thereby encouraging active participation in tourism activities or products, which aids in establishing a close relationship with the destination and fostering emotional attachment [50]. To some extent, tourists are more predisposed to experience flow. When the culture of the Village Basketball League aligns with the intrinsic needs or interests of tourists, a higher degree of involvement is likely to be achieved, resulting in an enhanced sense of cultural experience and improved concentration and enjoyment in tourism activities. Therefore, the following hypothesis is presented in this paper:

*H*5: Tourists’ involvement plays a positive moderating role in tourism cultural experience and flow experience.

**Fig 1:**
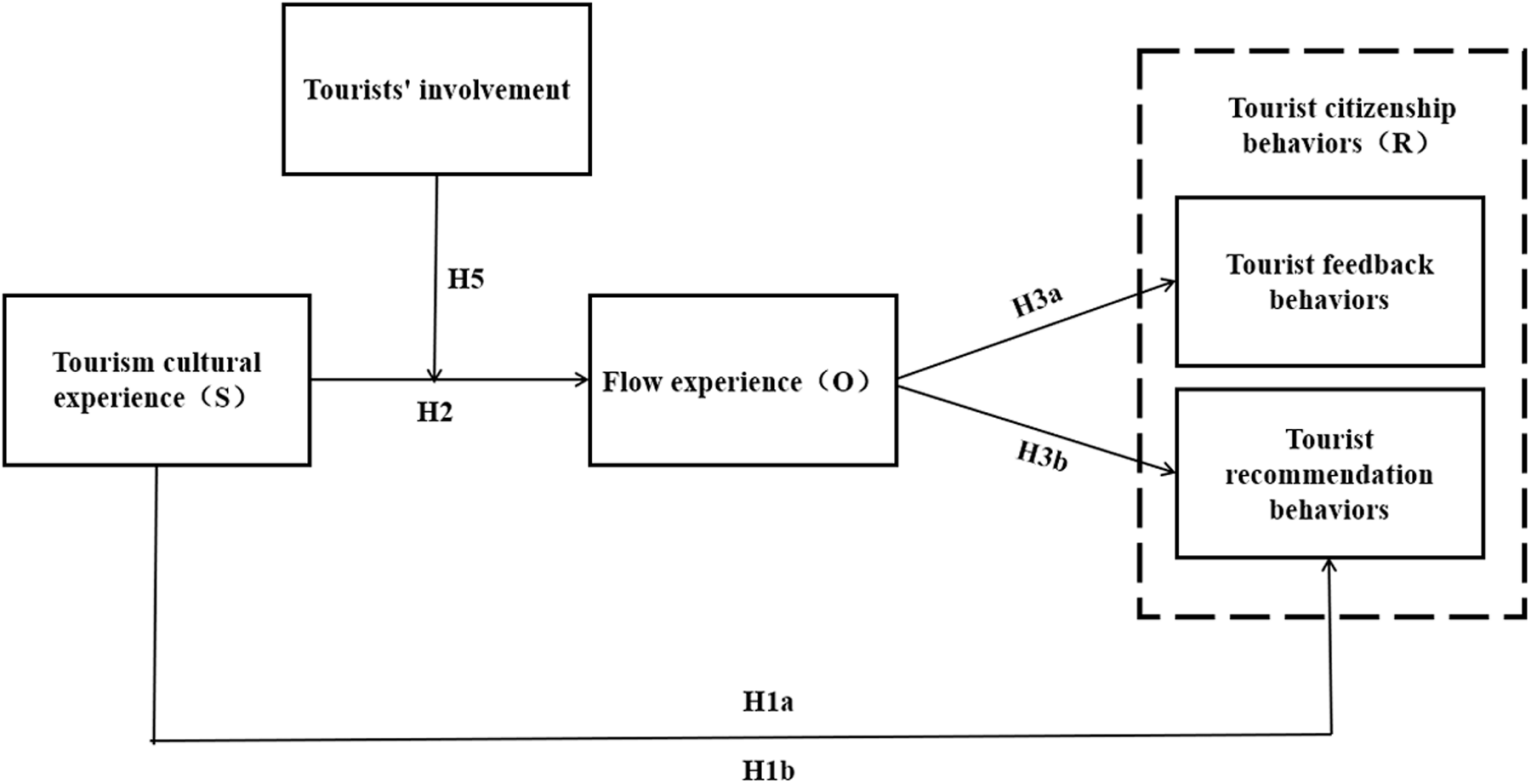
Theoretical model.

Based on the aforementioned literature review and research hypothesis, this study focused on the event scene of the Village Basketball League and developed a theoretical model to explore the relationships among tourism cultural experience, flow experience, tourists’ involvement, and tourist citizenship behaviors, as illustrated in Fig 1.

## 3 Research method

### 3.1 Study area

**Fig 2:**
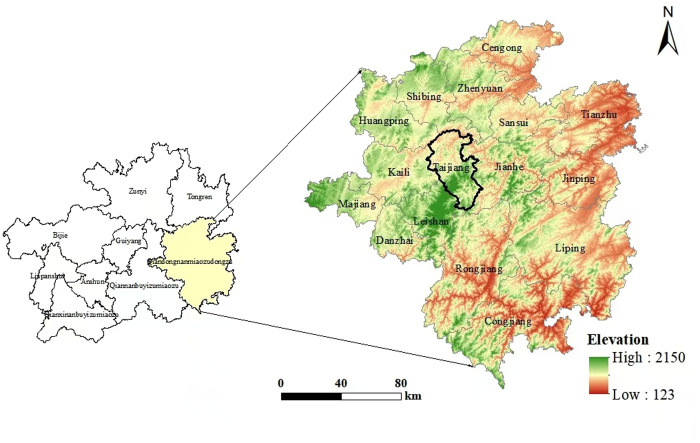
Location map of the study area.

This paper selects Taipan Village in Taijiang County, Guizhou Province, China as the case study (Fig 2). In July 2022, the “Village Basketball League” basketball tournament held in Guizhou attracted significant attention. Taipan Village’s “June 6th Eating New Festival” basketball game is recognized as the predecessor of the “Village Basketball League,” and the tradition of holding a basketball game in the Eating New Festival has persisted for decades. “Village Basketball League” has attracted a large number of tourists to watch and participate in the competition, which has promoted the development of local tourism. According to data provided by Taijiang County, during the 2022 “Village Basketball League” competition, the county received 681,900 tourists and generated a comprehensive tourism income of 95.16 million yuan. Additionally, during the three days of the “Village Basketball League” finals in 2023, the number of tourists in the county reached 181,900, the comprehensive tourism income amounted to 55.16 million yuan, and tourism bookings in Qiandongnan increased by 140% year-on-year. “Village Basketball League” is not only a sports activity but also serves as a vivid practice of integrating culture and tourism. Through the festival sports culture, basketball fans from across the country are brought together. During the “Village Basketball League” competition, villagers wear local traditional ethnic costumes and perform songs and dances to showcase their local characteristics, allowing tourists to experience local folk festival activities, understand the national culture behind intangible cultural heritage products such as silver jewelry and batik, and appreciate the charm of traditional culture. The concentrated presentation of these cultural elements elevates the “Village Basketball League” beyond a mere sports event, transforming it into a multidimensional exhibition space for Miao culture. The modern transformation of this traditional festival not only preserves cultural authenticity but also broadens its audience through competitive entertainment, emerging as an innovative paradigm for enhancing tourism experiences with cultural depth. Although current sports events have gradually spread across the country, Taijiang County, known for its long history of basketball culture, rich and diverse national culture, and significant tourism absorption effect, remains the most typical and representative case, aiding this study in understanding the characteristics of tourist behavior in recreational tourism.

### 3.2 Measurement and questionnaire design

The questionnaire used in this study is divided into two parts: the first part consists of four scales: tourism cultural experience, flow experience, tourist involvement, and tourist citizenship behavior. The questionnaires in this section refer to established scales utilized by both domestic and international scholars and have been modified to incorporate the relevant content of the “Village Basketball League” in Taijiang County. The tourism cultural experience is based on the research conducted by Wang et al. [51], which includes four items; the measurement of flow experience is derived from the research of Liu et al. [37], which comprises three items. The items related to tourist involvement primarily refer to the scale developed by Suhartanto et al. [52], which consists of three items. Tourist citizenship behaviors are divided into two subscales: tourist feedback behaviors and tourist recommendation behaviors. Specifically, tourist feedback behaviors are based on the scale content developed by Wang [53] and Chen [54], which includes four items, while tourist recommendation behaviors are based on the scale content developed by Groth [29], which consists of three items. The Likert 7-level scale was employed for all measurement items in the questionnaire. The second part includes basic information about the sample, such as gender, age, and educational background.

### 3.3 Data collection and sample characteristics

Before the formal investigation, 50 questionnaires were distributed on an online platform for pre-investigation purposes to assess the reasonableness of the content and arrangement of the questions. Following minor modifications based on the data results, the formal questionnaires were distributed between October 25 and October 29, 2023. The research sample for this study consists of tourists who have travel experience with the “Village Basketball League.” A random sampling method was employed to collect data over five consecutive days at the event site in Taijiang County. The survey covered the core activity area of the “Village Basketball League,” and data was collected during times of high tourist density to enhance representativeness. Recognizing that tourists vary significantly in cultural distance, consumption power, travel motivation, and behavior due to differences in age, occupation, and region, the study established data collection sites in cultural experience areas, competition core areas, and catering areas. This approach was designed to capture tourist behaviors in diverse scenarios. Meanwhile, daily statistics were collected, and efforts to recruit participants on-site were intensified for groups with low coverage. Stratified criteria were applied to filter the data, ensuring diversity in the sample in terms of region, age, and occupation. Researchers approached visitors to explain the study’s purpose and invited them to voluntarily participate in the survey. Respondents provided written informed consent before completing the questionnaire. This study was reviewed and approved by the Ethics Review Committee of the College of Digital Economy at Fujian Agriculture and Forestry University, with the ethical approval number [FAFUaxcxy-2023-11]. A total of 400 questionnaires were issued face to face, 86 invalid questionnaires were recovered and screened, and 314 valid questionnaires were obtained, with an effective recovery rate of 79%.

According to the statistical results of the sample (Table 1), the proportion of female tourists exceeded that of male tourists, reaching 66.6%. The predominant age groups are 18-29 years and 30-39 years, accounting for 88.9% of the sample, indicating that the tourists participating in “Village Basketball League” tourism are predominantly young individuals. This is likely because the “Village Basketball League” offers a novel tourism experience that aligns with the interests of young individuals in sports and allows them to experience diverse rural cultures and lifestyles. In contrast to the fast pace of urban life, this experience is highly attractive to young individuals. Regarding educational background, junior college and undergraduate students constitute the largest group, accounting for 70.7%. In terms of monthly income, 54.1% of the sample reported an income exceeding CNY 8000. Regarding occupation, the largest group comprises corporate employees and freelancers, accounting for approximately 68.8%. Additionally, a majority of the respondents originated from other provinces and cities, accounting for 83.1%. This may be attributed to the fact that for local residents of Guizhou Province and Taijiang County, the “Village Basketball League” is a common activity, leading them to prefer other tourist destinations or activities for leisure time rather than limiting themselves to local tourism. Tourists from other provinces and cities may be more inclined to seek new travel experiences; thus, the majority of “Village Basketball League” tourists in Taijiang County are from outside the province.

**Table 1:** Demographic characteristics of the sample.

Items	Categories	Frequency	Percent(%)
Gender	Male	105	33.4
	Female	209	66.6
Age	18-29	118	37.6
	30-39	161	51.3
	40-49	25	8.0
	50-59	9	2.9
	60 or over	1	0.3
Educational background	Senior high school/technical secondary school and below	13	4.1
	Junior college/undergraduate	222	70.7
	Master’s degree or above	79	25.2
Monthly income(RMB)	2000 or below	35	11.1
	2001-4000	30	9.6
	4001-6000	27	8.6
	6001-8000	52	16.6
	8000 Over	170	54.1
Occupation	Student	52	16.6
	Civil servant/public institution personnel	36	11.5
	Corporate employee/freelancer	216	68.8
	Other occupations	10	3.2
Region	Taijiang County, Guizhou Province	13	4.1
	Guizhou Province (excluding Taijiang County)	40	12.7
	Other provinces and cities	261	83.1

### 3.4 Data analysis

The partial least squares structural equation model (PLS-SEM) was employed to analyze the collected data. In comparison to traditional CB-SEM, PLS-SEM is more conducive to the analysis of small samples and complex models [55,56]. Additionally, since PLS-SEM does not require data to conform to a normal distribution [57], it is suitable for the survey data collected in this study. Therefore, SmartPLS 4.0 software is utilized for data analysis.

## 4 Result analysis

### 4.1 Measurement model analysis

PLS-SEM was employed to analyze the measurement model, and the results are presented in Table 2. The standard factor loadings for each variable ranged from 0.609 to 0.851, all exceeding 0.6. The Cronbach’s α values for all variables were above 0.7, and the composite reliability (CR) ranged from 0.820 to 0.871, all within the acceptable range. Moreover, the average variance extracted (AVE) values were all above 0.5. These results indicate that the five variables in this study exhibited good internal consistency and convergent validity [58].

**Table 2:** Measurement model verification.

	Item descriptions	Standard factor loadings	Cronbach’s α	CR	AVE
Tourism cultural experience (CE)	CE1 Through the “Village Basketball League” travel experience, I feel the local unique local culture	0.830	0.722	0.828	0.549
	CE2 Through the “Village Basketball League” travel experience, I felt the unique lifestyle of local people	0.774			
	CE3 Through the “Village Basketball League” travel experience, I feel the local characteristics of folk customs	0.733			
	CE4 Through the “Village Basketball League” travel experience, I felt the local people’s love for basketball	0.609			
Tourists’ involvement (TI)	TI1 I’m interested in “Village Basketball League” Tours	0.832	0.736	0.850	0.655
	TI2 “Village Basketball League” Tour is a very important activity for me	0.778			
	TI3 “Village Basketball League” Tour is very relaxing for me	0.817			
Flow experience (FE)	FE1 While watching the “Village Basketball League” events, I felt that time passed very quickly	0.821	0.734	0.849	0.652
	FE2 While watching the “Village Basketball League” events, my focus was entirely on the event	0.788			
	FE3 While watching the “Village Basketball League” events, I found the travel experience very interesting	0.812			
Tourist feedback behaviors (FB)	FB1 I will fill in the “village Basketball League” tourist satisfaction survey carefully	0.675	0.707	0.820	0.534
	FB2 If there is any defect in the tourism facilities or tourism services of “Village Basketball League”, I will take the initiative to inform the service provider	0.680			
	FB3 When I receive good service from “Village Basketball League” local staff, I thank and inform the relevant organization	0.739			
	FB4 I am willing to offer my suggestions to the local authorities to improve the quality of services and products	0.819			
Tourist recommendation behaviors (RB)	RB1 I would rather go to the “village Basketball League” than any other tourist destination	0.851	0.778	0.871	0.692
	RB2 I would encourage others to visit the “village Basketball League” tourist destination	0.821			
	RB3 I would recommend the “village Basketball League” on social networking sites	0.824			

In addition, according to the criteria of the Fornell-Larcker method, the square root of the average variance extracted (AVE) for each variable is greater than the correlation coefficients between the corresponding variables [55]. As shown in Table 3, the results of this study meet these requirements, indicating that all variables possess good discriminative validity.

**Table 3:** Discriminative validity analysis.

	Tourism cultural experience	Tourists’ involvement	Flow experience	Tourist feedback behaviors	Tourist recommendation behaviors
Tourism cultural experience	0.741				
Tourists’ involvement	0.712	0.809			
Flow experience	0.728	0.745	0.807		
Tourist feedback behaviors	0.650	0.639	0.684	0.731	
Tourist recommendation behaviors	0.711	0.742	0.742	0.686	0.832

### 4.2 Structural model test

In this study, Smart PLS 4.0 was utilized to test the structural model, and the results were subsequently analyzed. The R² value is employed to measure the explanatory ability of the model and serves as an important indicator of PLS-SEM. The results of this study indicate that the R² values for flow experience, tourist feedback behaviors, and tourist recommendation behaviors are 0.645, 0.517, and 0.644, respectively, all exceeding 0.3, which indicates a good explanatory level of the model [59]. Additionally, the prediction coefficients Q² for these three variables are 0.400, 0.261, and 0.427, all of which exceed 0, indicating that the model possesses strong forecasting ability [60]. According to Hu and Bentler, an SRMR value of less than 0.08 indicates a good model fit [61]. In this study, the SRMR value is 0.079, suggesting that the structural model demonstrates a satisfactory fit.

As indicated by the results in Table 4, the tourism cultural experience has significant positive effects on tourists’ feedback behaviors (β=0.324, *p*<0.001), tourists’ recommendation behaviors (β=0.279, *p*<0.001) and flow experience (β=0.315, *p*<0.001), indicating that hypotheses H1a, H1b, and H2 are supported. The flow experience has significant positive effects on tourists’ feedback behaviors (β=0.448, *p*<0.001) and tourists’ recommendation behaviors (β=0.364, *p*<0.001), indicating that hypotheses H3a and H3b are supported. This suggests that the flow experience is an important factor in the development of tourist citizenship behaviors. The stronger the sense of flow experience, the easier it becomes for tourists to exhibit citizenship behaviors that are favorable to the tourist destination, such as feedback behaviors and recommendation behaviors.

**Table 4:** Hypothesis testing results.

Research hypotheses	Path coefficient	Standard deviation	t-value	Results
H1a:TY→FK	0.324***	0.087	3.708	Supported
H1b:TY→TJ	0.279***	0.061	4.605	Supported
H2:TY→XL	0.315***	0.065	4.856	Supported
H3a:XL→FK	0.448***	0.082	5.441	Supported
H3b:XL→TJ	0.364***	0.063	5.824	Supported

***p<0.001, **p<0.01, *p<0.05.

### 4.3 Mediating effect test

The PLS bootstrap repeated sampling method (5000 times sampling) was utilized to test the mediating relationship, and the results are presented in Table 5. A significant mediating path is observed for flow experience between tourist cultural experience and tourist feedback behaviors (β=0.141, *p*<0.01), indicating that hypothesis H4a are supported; a significant mediating path is also observed for flow experience between tourist cultural experience and tourist recommendation behavior (β=0.115, *p*<0.001), indicating that hypothesis H4b are supported. This indicates that the tourism cultural experience, as an antecedent variable, can indirectly promote tourists’ feedback and recommendation behaviors through the mediating role of flow experience. Therefore, to enhance tourists’ civic behavior, attention should be paid not only to the cultural experience of tourists at the tourist destination but also to improving their flow experience during tourism.

**Table 5:** Results of mediating effect test.

Research hypotheses	Path coefficient	Standard deviation	t-value	Results
H4a:TY→XL→FK	0.141**	0.044	3.220	Supported
H4b:TY→XL→TJ	0.115***	0.027	4.271	Supported

***p<0.001, **p<0.01, *p<0.05.

### 4.4 Moderating effect test

The research results indicate that the interaction term between tourist cultural experience and tourist involvement has a significant negative effect on flow experience (β=-0.040, *p*<0.05); that is, tourist involvement weakens the positive influence of tourist cultural experience on flow experience. The greater the involvement of tourists in “Village Basketball League” tourism, the weaker the influence of tourist cultural experience on flow experience. This finding is inconsistent with the hypothesis, indicating that hypothesis H5 is not supported. This indicates that the “Village Basketball League” competition failed to provide tourists with an impressive and engaging tourism experience, resulting in tourists gradually losing interest after participating in local tourism activities and being unable to immerse themselves in them. The discrepancy between the empirical results of this study and those of previous studies may be attributed to the focus of traditional tourism involvement research. Typically, such studies concentrate on static cultural landscapes, including museums and historical districts [62], or on food [63] and products [64] within tourist destinations, where tourists gradually become immersed during their visits. Conversely, in dynamic sports events or folk activities, highly involved tourists may become excessively focused on game results or team performance. This heightened focus can diminish their depth perception of cultural symbols, increasing psychological load while reducing immersion [65]. Moreover, high involvement is often accompanied by elevated expectations. When there is a significant disparity between the actual cultural experience provided and tourists’ expectations, the moderating effect of involvement may shift from positive to negative. For instance, some tourists develop strong expectations for the culture of the “Village Basketball League” due to social media promotion, but the actual activities may be relatively simple with limited participatory opportunities. In such cases, tourists with high involvement are more likely to perceive a lack of cultural authenticity [66], leading to cognitive dissonance and a diminished flow experience. When tourism experiences fail to meet expectations, negative emotional reactions are significantly amplified [67].

## 5 Conclusions and implications

### 5.1 Research conclusions

Based on the theoretical framework of stimulus-body-response, this study begins with the external stimulus of tourism cultural experience, introduces the variables of tourist involvement and flow experience, and constructs a model to examine the relationship between tourism cultural experience and tourists’ civic behavior. The main conclusions are presented as follows: (1)The cultural experience of tourism has a positive impact on tourists’ civic behavior. The two dimensions of tourist citizenship behaviors—feedback behaviors and recommendation behaviors—are significantly and positively influenced by tourism cultural experience. This indicates that in the context of “Village Basketball League” tourism, unique cultural experiences prompt tourists to exhibit civic behavior that supports the tourism destination. (2)The flow experience serves as a mediating factor between tourism cultural experience and tourist citizenship behaviors. Within the context of “Village Basketball League” the tourism cultural experience, flow experience, and tourist citizenship behaviors can be conceptualized as forming a “stimulus-organism-response” relationship chain, and tourism cultural experience can promote tourist citizenship behaviors such as feedback and recommendation through flow experience. (3)The relationship between tourist cultural experience and flow experience is regulated by the degree of tourists’ involvement. Specifically, in the “village Basketball League” tourism context, tourists’ involvement can inhibit the positive impact of tourism cultural experience on flow experience, and play a negative moderating role.

### 5.2 Theoretical implications

(1) This paper incorporates the SOR theoretical framework into the study of the integration of culture, sports, and tourism, thereby transcending the limitations of traditional tourism behavior theories that often focus on a single field. This approach extends the application boundaries of SOR theory. While existing research on tourist experience and behavior has yielded substantial theoretical insights [68,69], scholars acknowledge that the sense of experience influences tourist behavior. However, there is still a lack of comprehensive exploration of tourist experience and behavior within the context of cultural, sports, and tourism integration. With the rise of cultural tourism, more destinations are leveraging the Internet to market their cultural offerings [70], and attract tourists seeking immersive experiences. Activities such as sports events and folk festivals are especially popular among tourists. On the one hand, this study further confirms the applicability of previous studies. The “Village Basketball League” in Taijiang County integrates culture and sports, attracting mostly tourists interested in local festival culture and basketball culture. Tourists perceive and immerse themselves in the destination’s culture, feeling satisfied with the cultural experience process. Consequently, they are likely to engage in civic actions, such as providing feedback on local facilities and services or recommending the destination to relatives and friends. On the other hand, this study adds new insights to the SOR theoretical framework. First, tourists’ involvement is innovatively introduced as a moderating variable for the internal state of the organism, providing a novel perspective for subsequent research. Secondly, while previous research frameworks typically focus on a single behavioral variable, this paper includes tourists’ feedback and recommendation behaviors as outcome variables, thereby extending the study of the SOR theoretical framework.

(2) This study confirms that cultural experiences at tourist destinations can be transmitted through flow experiences, systematically revealing the mechanisms by which cultural experiences influence tourist feedback and recommendation behaviors within the context of cultural and sports tourism. Previous research that uses flow experience as an intermediary variable predominantly considers electronic products, website functionalities, and social media marketing tools—such as short videos and live broadcasts—as antecedent variables [71–73], while focusing on tourists’ behaviors and attitudes as outcome variables. However, there has been limited exploration of the role of flow experience in the context of sports events, folk culture tourism experiences, and tourist citizenship behaviors. By embedding the mediating effect of flow experience into the culture-driven context, this study reconstructs the “experience-psychology-behavior” chain through a cultural immersion lens, offering empirical validation for flow theory’s cross-domain applicability in cultural tourism settings. Meanwhile, this study enhances the understanding of how cultural factors elucidate tourists’ altruistic behaviors. It not only deconstructs the internal psychological mechanisms that transform cultural symbols into tourists’ spontaneous communication behaviors but also reveals the catalytic effect of immersive cultural experiences on transforming tourists’ roles from “bystander” to “co-creator” during the integration of cultural tourism. This addresses a gap in traditional tourist behavior research, which often overlooks the path of cultural psychological transformation [74,75]. Therefore, introducing flow experience as an intermediary variable in this study not only expands its theoretical application but also enhances tourists’ understanding of the mechanisms underlying the distinctive culture of the tourist destination.

(3) This study uncovers the negative moderating effect of tourists’ involvement within the integrated context of culture, sports, and tourism, challenging the conventional perspective of involvement theory and offering valuable insights for subsequent research on tourist behavior. Current studies on tourism involvement generally posit that tourism involvement exerts a positive influence. For example, Muñoz-Leiva et al., in their study on the effectiveness of e-tourism advertising, observed that the impact of visual attention from banner advertisements on individual memory is positively correlated with the level of involvement, thereby amplifying the advertising effect [76]. Similarly, Pérez Benegas et al. found in their research that the level of involvement positively mediates the relationship between the cognitive and emotional dimensions of customer engagement and further positively regulates the connection between these dimensions and customer loyalty on social media platforms [77]. However, this study discovered that within the context of the deep integration of dynamic sports events and national culture, such as the “Village Basketball League,” tourists with high involvement concentrated excessively on the competitive aspects of the event, thereby diminishing their immersive perception of cultural symbols. This resulted in the inhibition of the influence of the tourism cultural experience on flow experience. This study confirms the scene sensitivity of involvement levels and proposes that the direction of the effect of involvement on tourists’ psychology depends on the degree of alignment between goal orientation and cultural experience. In the context of integrating culture, sports, and tourism, if tourists with high involvement are primarily motivated by the pursuit of game experience, they may overlook the profound value of cultural experience. This can lead to a mismatch of psychological resources and a disruption of immersion. This finding not only expands the theoretical boundaries of tourist behavior research but also challenges the one-way cognitive framework of tourists’ involvement, providing deeper theoretical guidance for the sustainable development of cultural and tourism integration.

### 5.3 Managerial implications

The attitudes and citizenship behaviors of tourists have always been concerns for tourist destinations, governments, and related organizations, as they are linked to the sustainable development of these destinations. Therefore, in today’s increasingly competitive tourism environment, the stimulation of tourists’ citizenship behaviors warrants attention and consideration. Based on the aforementioned empirical results and the current situation, this study presents the following implications:

Firstly, regional cultural characteristics should be leveraged to develop cultural tourism activities and products that stimulate tourists’ flow experience. Specifically, local cultural elements should be integrated into tourism activities to enhance the quality of the tourism experience. First, the government should provide funding and policy support to collaboratively develop experience workshops, such as Miao silver jewelry making and tie-dyeing techniques, with inheritors of intangible cultural heritage technology. Concurrently, the community should be responsible for implementing these activities, enabling tourists to engage with local folk culture and savor local specialty foods, thereby forming a comprehensive value loop. This approach aims to enhance tourists’ cultural perceptions, facilitate the development of rural tourism, and ultimately achieve cooperation and mutual benefits among tourists, the government, and tourist destinations. Second, create a series of experiential products centered around the “Village Basketball League” cultural brand. Develop the arena into an exhibition area showcasing national and local cultures, and establish cultural experience and festival activity areas surrounding the village. Miao cultural elements are intricately woven into the basketball competition, including the Miao ancient song duet ceremony before the game, Lusheng dance performances during halftime, and workshops on Miao silver ornaments or batik handicrafts organized post-game. Through folk customs and agricultural product exhibitions, tourists will gain a deeper understanding of rural culture while enjoying the events, thereby enhancing their emotional connection and engagement with the tourist destination. Third, systematically organizing local cultural resources and utilizing AR technology to restore cultural scenes is essential. Creating online and offline links to a “cultural database” and establishing an AR intelligent navigation system around the stadium will allow tourists to trigger scenes such as the “Village Basketball League” folk festival story, Miao clothing and song and dance, and intangible cultural heritage skills interpretation via mobile phone scanning. This integration enhances the immersive experience by combining virtual and real elements. In short, it is necessary to pay attention to the immersion experience process of tourists, create a good cultural atmosphere and high-quality cultural products to attract tourists, so that tourists can have a profound flow experience in experiencing festival activities and folk crafts, and then be willing to give feedback on the development of the tourism destination and recommend the tourism destination to others.

Secondly, it is necessary to provide tourism activities for tourists in different categories to play the role of tourists’ involvement. A singular form of activity is insufficient to sustain long-term tourist interest. Therefore, it’s crucial to integrate the culture of the tourist destination with its history, folk customs, festivals, and other elements to develop distinctive tourism projects. First, invite sports stars to engage in public interest activities to boost the event’s appeal and social impact. Collaborate with authoritative media and utilize digital channels such as short video platforms and live broadcasts for targeted marketing, enhancing interaction with potential tourists and communication reach. Second, analyze tourist psychology to cater to personalized tourism needs and interests of diverse groups, innovate cultural activity forms and content, and offer varied activities for different tourist types and age groups. For tourists with high involvement, provide opportunities for deep engagement, such as temporary team participation and interactive training with local players. For those with lower involvement, offer experiences in intangible culture and local food tasting to prevent information overload and reduce psychological burden. Third, improve local infrastructure by establishing “cultural stations” in areas with high cultural activity concentration, offering free drinking water, charging stations, and multilingual tour guide systems to increase tourists’ happiness and satisfaction during their visit. Additionally, tourism destination managers should focus on fostering interaction and participation in activities, enabling tourists to fully experience local culture, playing the positive regulating role of tourists’ involvement, and enhancing the influence mechanism of cultural experience.

Finally, develop a comprehensive tourist experience platform to ensure the long-term attraction of tourism destinations. Over time, it is crucial for these destinations to sustain their appeal and maintain visitor interest through continuous content innovation, community collaboration, and technological empowerment. First, establish different cultural themes annually and launch exclusive activities alongside “Village Basketball League” competitions. Collaborate with local communities to host regular “Village Basketball League” cultural festivals, integrating ethnic traditions to create a featured activity that combines sports and folk culture. Second, encourage tourists and villagers to collectively build cultural memories by offering small Miao language classes and creating cultural wall graffiti, thereby enhancing emotional connections. Cultivate local cultural ambassadors who can continuously promote “Village Basketball League” and cultural integration content via live broadcasts and short videos, maintaining online engagement. Third, focus on “sports + culture” as the core for coordinated development across multiple industries. Integrate skills like Miao silver jewelry crafting, Miao embroidery, and batik to develop basketball-themed cultural and creative products, such as silver basketball pendants and Miao embroidery T-shirts with basketball motifs as event souvenirs. Package local specialty agricultural products as “Village Basketball League” competition cheering products and sell them both around the arena and on online platforms. Additionally, establish a “Village Basketball League” tourism big data platform to analyze tourist behavior, consumption, and feedback, providing a foundation for optimizing the industrial chain.

### 5.4 Research limitations and future research

First, while this paper utilizes offline questionnaires to enrich the sample data, the sample size remains significantly smaller than the total population of “Village Basketball League” tourists, which may cause certain biases. Future research will consider web text analysis and expand the sample size to address this limitation. Second, this paper takes Taijiang County, the “Village Basketball League” cultural tourism destination, as the case study. This singular focus means that the findings may not be applicable to other tourist destinations. Future research could explore a variety of cultural tourism destinations, such as the Tulou in Fujian Province and the Ice and Snow World in Harbin, to enhance the universal applicability of the research model. Finally, this paper only investigates the impact of cultural and flow experiences on tourist behavior. In reality, the factors influencing tourist behavior are more complex and varied. Therefore, future research could expand to include additional variables, such as cultural distance and emotional connection, to gain a more comprehensive understanding of tourist behavior.

## Supporting information

S1 File Data. (XLSX)

## References

[1] Kim H, Chen JS. The Memorable Travel Experience and Its Reminiscence Functions. Journal of Travel Research. 2018;58(4):637–49. doi: 10.1177/0047287518772366

[2] UNWTO. Big Data in Cultural Tourism: Building Sustainability and Enhancing Competitiveness. Madrid: World Tourism Organization (UNWTO); 2021.

[3] Salem IE, El‐Said OA, El Gamil R, Afifi MF, Attallah NF. How tourism cultural events influence multicultural competence, tourism destination image, and visit intentions: evidence from the Pharaohs’ golden parade. Int J Tour Res. 2024;26(5):e2740. 10.1002/jtr.2740

[4] Stanovčić T, Manojlović M, Perovic D. The Relationship between Cultural Tourist Experience and Recommendation Intention: Empirical Evidence from Montenegr. Sustainability. 2021;13(23):13144. doi: 10.3390/su132313144

[5] Cheng Z, Chen X. The Effect of Tourism Experience on Tourists’ Environmentally Responsible Behavior at Cultural Heritage Sites: The Mediating Role of Cultural Attachment. Sustainability. 2022;14(1):565. doi: 10.3390/su14010565

[6] Kong Y-Q, Kwon Y-J, Girish VG, Lee C-K, Reisinger Y. The impact of destination brand experience on arousal, memorable tourism experience, and revisit intention: Gyeongbok palace night tour. Asia Pacific Journal of Tourism Research. 2024;29(9):1063–78. doi: 10.1080/10941665.2024.2367490

[7] Lin J, Kang Y, Hong L, Huang Y. Can cultural tourism experience enhance cultural confidence? The evidence from Qingyuan Mountain. Front Psychol. 2022;13:1063569. doi: 10.3389/fpsyg.2022.1063569 36600717 PMC9806236

[8] Bai WB, Wang JJ, Wong JWC, Han XH, Guo Y. The soundscape and tourism experience in rural destinations: an empirical investigation from Shawan Ancient Town. Humanit Soc Sci Commun. 2024;11(1):1–12. 10.1057/s41599-024-02997-4

[9] Tabaeeian RA, Yazdi A, Mokhtari N, Khoshfetrat A. Host-tourist interaction, revisit intention and memorable tourism experience through relationship quality and perceived service quality in ecotourism. Journal of Ecotourism. 2022;22(3):406–29. doi: 10.1080/14724049.2022.2046759

[10] Hosany S, Prayag G, Van Der Veen R, Huang S, Deesilatham S. Mediating Effects of Place Attachment and Satisfaction on the Relationship between Tourists’ Emotions and Intention to Recommend. Journal of Travel Research. 2016;56(8):1079–93. doi: 10.1177/0047287516678088

[11] Shafiee MM, Tabaeeian RA, Khoshfetrat A. Tourist engagement and citizenship behavior: The mediating role of relationship quality in the hotel industry. Tour Hosp Res. 2020;20(4):481–92. 10.1177/1467358420914373

[12] Bi J, Wang B, Lu F. Does Host-Guest Interaction Stimulate Tourists’ Citizenship Behavior? A Combination of Social Exchange Theory and Cognitive Appraisal Theory. Forests. 2024;15(7):1156. doi: 10.3390/f15071156

[13] Zhang H, Cheng Z, Chen X. How Destination Social Responsibility Affects Tourist Citizenship Behavior at Cultural Heritage Sites? Mediating Roles of Destination Reputation and Destination Identification. Sustainability. 2022;14(11):6772. doi: 10.3390/su14116772

[14] Rather RA, Raisinghani M, Gligor D, Parrey SH, Russo I, Bozkurt S. Examining tourist citizenship behaviors through affective, cognitive, behavioral engagement and reputation: Symmetrical and asymmetrical approaches. J Retail Consum Serv. 2023;75:103451. 10.1016/j.jretconser.2023.103451

[15] Zhou G, Liu Y, Hu J, Cao X. The effect of tourist-to-tourist interaction on tourists’ behavior: The mediating effects of positive emotions and memorable tourism experiences. J Hosp Tour Manag. 2023;55:161–8. 10.1016/j.jhtm.2023.03.005

[16] Han J, Lee M, Hwang Y-S. Tourists’ Environmentally Responsible Behavior in Response to Climate Change and Tourist Experiences in Nature-Based Tourism. Sustainability. 2016;8(7):644. doi: 10.3390/su8070644

[17] Mehrabian A, Russell JA. An approach to environmental psychology. Cambridge: MIT Press; 1974.

[18] Ye C, Zheng R, Li L. The effect of visual and interactive features of tourism live streaming on tourism consumers’ willingness to participate. Asia Pacific Journal of Tourism Research. 2022;27(5):506–25. doi: 10.1080/10941665.2022.2091940

[19] Hew JJ, Leong LY, Tan GWH. Mobile social tourism shopping: A dual-stage analysis of a multi-mediation model. Tour Manag. 2018;66:121–39. 10.1016/j.tourman.2017.10.005

[20] Kim MJ, Lee C-K, Jung T. Exploring Consumer Behavior in Virtual Reality Tourism Using an Extended Stimulus-Organism-Response Model. Journal of Travel Research. 2018;59(1):69–89. doi: 10.1177/0047287518818915

[21] Jiang J. The role of natural soundscape in nature-based tourism experience: an extension of the stimulus–organism–response model. Current Issues in Tourism. 2020;25(5):707–26. doi: 10.1080/13683500.2020.1859995

[22] Ouyang L, Zhang S, Zhu S, Liu Z, Li J. Digital Technology in Tourism Dance Performance: Exploring the Influence of Tourists’ Flow Experience and Meaningful Experience on Revisit Intention. IEEE Access. 2024;12:46347–61. doi: 10.1109/access.2024.3382291

[23] Yang W, Chen Q, Huang X, Xie M, Guo Q. How do aesthetics and tourist involvement influence cultural identity in heritage tourism? The mediating role of mental experience. Front Psychol. 2022;13:990030. doi: 10.3389/fpsyg.2022.990030 36389488 PMC9650545

[24] Yadav N, Verma S, Chikhalkar RD. eWOM, destination preference and consumer involvement – a stimulus-organism-response (SOR) lens. Tour Rev. 2022;77(4):1135–52. 10.1108/tr-10-2020-0506

[25] Ye D, Cho D, Liu F. Investigating the impact of virtual tourism on travel intention during the post-COVID-19 era: evidence from China. Univers Access Inf Soc. 2022:1–17. 10.1007/s10209-022-00952-1 PMC9707137 36466580

[26] Liu H, Park KS, Wei Y. An extended stimulus-organism-response model of Hanfu experience in cultural heritage tourism. J Vacat Mark. 2024;30(2):288–310. 10.1177/13567667221135197

[27] Wen H, Wong IA, Fan Y, Leong AMW. When festitivity meets heritage site: co-developed experience through the lens of situated cognition. Journal of Travel & Tourism Marketing. 2022;39(5):516–33. doi: 10.1080/10548408.2022.2148039

[28] Chen S, Tian Y, Pei S. Technological Use from the Perspective of Cultural Heritage Environment: Augmented Reality Technology and Formation Mechanism of Heritage-Responsibility Behaviors of Tourists. Sustainability. 2024;16(18):8261. doi: 10.3390/su16188261

[29] Groth M. Customers as good soldiers: Examining citizenship behaviors in internet service deliveries. J Manag. 2005;31(1):7–27. 10.1177/0149206304271375

[30] Liu JS, Tsaur SH. We Are in the Same Boat: Tourist Citizenship Behaviors. Tour Manag. 2014;42(6):88–100. 10.1016/j.tourman.2013.11.001

[31] Liu L, Cui T, Wu J. Encouraging tourist citizenship behavior through resource uniqueness and service quality: The mediating role of emotions. J Vacat Mark. 2021;27(1):45–60.

[32] Kim J-H, Ritchie JRB, McCormick B. Development of a Scale to Measure Memorable Tourism Experiences. Journal of Travel Research. 2010;51(1):12–25. doi: 10.1177/0047287510385467

[33] Lee S, Jeon S, Kim D. The impact of tour quality and tourist satisfaction on tourist loyalty: The case of Chinese tourists in Korea. Tour Manag. 2011;32(5):1115–24.

[34] Cheng TM, Chen MT. Creative atmosphere in creative tourism destinations: conceptualizing and scale development. J Hosp Tour Res. 2023;47(3):590–615. 10.1177/10963480211012459

[35] Kim J-H. The Impact of Memorable Tourism Experiences on Loyalty Behaviors: The Mediating Effects of Destination Image and Satisfaction. Journal of Travel Research. 2017;57(7):856–70. doi: 10.1177/0047287517721369

[36] Xu S, Kim HJ, Liang M, Ryu K. Interrelationships between tourist involvement, tourist experience, and environmentally responsible behavior: a case study of Nansha Wetland Park, China. Journal of Travel & Tourism Marketing. 2018;35(7):856–68. doi: 10.1080/10548408.2018.1439429

[37] Liu X, Zhang L, Chen Q. The effects of tourism e-commerce live streaming features on consumer purchase intention: The mediating roles of flow experience and trust. Front Psychol. 2022;13:995129. doi: 10.3389/fpsyg.2022.995129 36092030 PMC9462463

[38] Li C, He J. Restorative Environmental Perception’s Influence on Post-Tour Behavior of Desert Off-Road Self-Driving Tourists: The Mediating Role of Flow Experience. Sustainability. 2023;15(17):12934. doi: 10.3390/su151712934

[39] Yang W, Zhang Y, Wang Y-C. Would Travel Experiences or Possessions Make People Happier? Journal of Travel Research. 2022;62(2):412–31. doi: 10.1177/00472875211064631

[40] Park S, Ahn D. Seeking Pleasure or Meaning? The Different Impacts of Hedonic and Eudaimonic Tourism Happiness on Tourists’ Life Satisfaction. Int J Environ Res Public Health. 2022;19(3):1162. doi: 10.3390/ijerph19031162 35162186 PMC8834700

[41] Wu TC, Lin YE, Wall G. A spectrum of indigenous tourism experiences as revealed through means-end chain analysis. Tour Manag. 2020;76:103969. 10.1016/j.tourman.2019.103969

[42] Akçakese A, Demirel M, Yolcu AF, Gümüş H, Ayhan C, Sarol H, et al. Nature relatedness, flow experience, and environmental behaviors in nature-based leisure activities. Front Psychol. 2024;15:1397148. doi: 10.3389/fpsyg.2024.1397148 38903476 PMC11189019

[43] Kim M, Thapa B. Perceived value and flow experience: Application in a nature-based tourism context. J Destin Mark Manag. 2018;8:373–84. 10.1016/j.jdmm.2017.08.002

[44] Back RM, Tasci AD, Milman A. Experiential consumption of a South African wine farm destination as an agritourism attraction. J Vacat Mark. 2020;26(1):57–72. 10.1177/1356766719858642

[45] Li C, He J. Restorative Environmental Perception’s Influence on Post-Tour Behavior of Desert Off-Road Self-Driving Tourists: The Mediating Role of Flow Experience. Sustainability. 2023;15(17):12934. doi: 10.3390/su151712934

[46] Karasakal S, Albayrak T. How to create flow experience during travel: The role of destination attributes. J Vacat Mark. 2022;28(3):303–18. 10.1177/13567667211053386

[47] Havitz ME, Dimanche F. Leisure Involvement Revisited: Conceptual Conundrums and Measurement Advances. Journal of Leisure Research. 1997;29(3):245–78. doi: 10.1080/00222216.1997.11949796

[48] Celsi RL, Olson JC. The role of involvement in attention and comprehension processes. J Consum Res. 1988;15(2):210–24.

[49] Huaman-Ramirez R. Self-congruity and domestic tourists’ attitude: the role of involvement and age. Anatolia. 2020;32(2):303–15. doi: 10.1080/13032917.2020.1869045

[50] Ji F, Wang F, Wu B. How does virtual tourism involvement impact the social education effect of cultural heritage? Journal of Destination Marketing & Management. 2023;28:100779. doi: 10.1016/j.jdmm.2023.100779

[51] Wang LG, Song W, Huang ZP. The influence of tourism perceived value and local identity on rural tourism preference behavior. J Northwest Norm Univ Nat Sci. 2023;59(04):94–101.

[52] Suhartanto D, Dean D, Nansuri R. The link between tourism involvement and service performance: Evidence from frontline retail employees. J Bus Res. 2018;83:130–7. 10.1016/j.jbusres.2017.10.039

[53] Wang JQ. Effect of host-guest interaction on tourist citizenship behavior of rural tourism in the context of high-quality development. Areal Res Dev. 2021;40(04):85–90.

[54] Chen X, Cheng ZF. A research on the influencing mechanism of red tourism tourist citizenship behavior from the experience value perspective. Tour Sci. 2021;35(06):50–66.

[55] Zhang S, Liang J, Ma Y, Chen Y, He Q. Destination image, nostalgic feeling, flow experience and agritourism: An empirical study of Yunling Tea Estate in Anxi, China. Front Psychol. 2022;13:954299. doi: 10.3389/fpsyg.2022.954299 36160530 PMC9496169

[56] Jr. JFH, Matthews LM, Matthews RL, Sarstedt M. PLS-SEM or CB-SEM: updated guidelines on which method to use. IJMDA. 2017;1(2):107. doi: 10.1504/ijmda.2017.087624

[57] Hair JF, Risher JJ, Sarstedt M, Ringle CM. When to use and how to report the results of PLS-SEM. EBR. 2019;31(1):2–24. doi: 10.1108/ebr-11-2018-0203

[58] Henseler J, Ringle CM, Sarstedt M. A new criterion for assessing discriminant validity in variance-based structural equation modeling. J Acad Mark Sci. 2015;43(1):115–35. 10.1007/s11747-014-0403-8

[59] Fornell C, Larcker DF. Structural equation models with unobservable variables and measurement error: algebra and statistics. J Mark Res. 1981;18(1):39–50. 10.1177/002224378101800313

[60] Sarstedt M, Ringle CM, Smith D. Partial least squares structural equation modeling (PLS-SEM): A useful tool for family business researchers. J Fam Bus Strateg. 2014;5(1):105–15. 10.1016/j.jfbs.2014.01.002

[61] Hu L, Bentler PM. Fit indices in covariance structure modeling: Sensitivity to underparameterized model misspecification. Psychological Methods. 1998;3(4):424–53. doi: 10.1037/1082-989x.3.4.424

[62] Yang W, Chen Q, Huang X, Xie M, Guo Q. How do aesthetics and tourist involvement influence cultural identity in heritage tourism? The mediating role of mental experience. Front Psychol. 2022;13:990030. doi: 10.3389/fpsyg.2022.990030 36389488 PMC9650545

[63] Chen J, Hsu F-C, Yan L, Lee HM, Zhang Y. Tourists’ Food Involvement, Place Attachment, and Destination Loyalty: The Moderating Role of Lifestyle. Behav Sci (Basel). 2023;13(8):629. doi: 10.3390/bs13080629 37622769 PMC10451238

[64] Chen XJ, Zhou M, Zhang CM. Investigating the impact of digital collectibles involvement on tourists’ on-sites visit intentions. Current Psychology. 2024;43(13):11651–62. 10.1007/s12144-023-05287-5

[65] Lee BC. The Effect of Gamification on Psychological and Behavioral Outcomes: Implications for Cruise Tourism Destinations. Sustainability. 2019;11(11):3002. doi: 10.3390/su11113002

[66] Li X, Wang C. Understanding the relationship between tourists’ perceptions of the authenticity of traditional village cultural landscapes and behavioural intentions, mediated by memorable tourism experiences and place attachment. Asia Pacific Journal of Tourism Research. 2023;28(3):254–73. doi: 10.1080/10941665.2023.2217959

[67] Deng Y, Wang X, Ma C. The impact of sports tourism motivation on tourist loyalty: The chain mediation effect of experience quality and tourist satisfaction. Int J Tour Res. 2024;26(5):e2769. 10.1002/jtr.2769

[68] Hofman K, Walters G, Hughes K. The effectiveness of virtual vs real-life marine tourism experiences in encouraging conservation behaviour. J Sustain Tour. 2022;30(4):742–66. 10.1080/09669582.2021.1884690

[69] Zhang J, Jin L, Pan X, Wang Y. Pro-Environmental Behavior of Tourists in Ecotourism Scenic Spots: The Promoting Role of Tourist Experience Quality in Place Attachment. Sustainability. 2024;16(20):8984. doi: 10.3390/su16208984

[70] Li J, Xie M, Yu M, Ahn Y. Understanding Tourists’ Social Networking Site (SNS) Intention with Regards to World Heritage Sites: The Role of Motivation and Overall Image. Sustainability. 2024;16(9):3538. doi: 10.3390/su16093538

[71] Jeon H, Ok C, Choi J. Destination marketing organization website visitors’ flow experience: an application of Plog’s model of personality. J Travel Tour Mark. 2017;35(4):397–409. doi: 10.1080/10548408.2017.1358234

[72] Kim D, Ko YJ. The impact of virtual reality (VR) technology on sport spectators’ flow experience and satisfaction. Comput Hum Behav. 2019;93:346–56. 10.1016/j.chb.2018.12.040

[73] Dong W-W, Wang Y-Q, Qin J. An empirical study on impulse consumption intention of livestreaming e-commerce: The mediating effect of flow experience and the moderating effect of time pressure. Front Psychol. 2023;13:1019024. doi: 10.3389/fpsyg.2022.1019024 36726500 PMC9885107

[74] Wang C, Zhang J, Xiao X, Sun F, Xiao M, Shi Q. Examining the dimensions and mechanisms of tourists’ environmental behavior: A theory of planned behavior approach. J Clean Prod. 2020;273:123007. 10.1016/j.jclepro.2020.123007

[75] Liu J, Li J, Jang S, Zhao Y. Understanding tourists’ environmentally responsible behavior at coastal tourism destinations. Marine Policy. 2022;143:105178. doi: 10.1016/j.marpol.2022.105178

[76] Muñoz-Leiva F, Liébana-Cabanillas F, Hernández-Méndez J. Etourism advertising effectiveness: banner type and engagement as moderators. J Serv Mark. 2018;32(4):462–75. 10.1108/jsm-01-2017-0039

[77] Perez Benegas JY, Zanfardini M. Customer engagement and loyalty: the moderating role of involvement. Eur J Manag Bus Econ. 2023;34(3):319–39. doi: 10.1108/ejmbe-03-2022-0074

